# Comprehensive comparative analysis of explainable deep learning model for differentiation of brucellar spondylitis and tuberculous spondylitis through MRI sequences

**DOI:** 10.1186/s40001-025-03731-9

**Published:** 2025-12-24

**Authors:** Parhat Yasin, Abudouresuli Tuersun, Anuar Ashir, Yerlan Makhambetov, Jie Sheng, Xinghua Song

**Affiliations:** 1 Department of Spine Surgery, The Sixth Affiliated Hospital of Xinjiang Medical University, Ürümqi, 830000 Xinjiang People’s Republic of China; 2 Xinjiang Key Laboratory of Artificial Intelligence Assisted Imaging Diagnosis, Department of Radiology, The First People’s Hospital of Kashi Prefecture, Kashi, 844000 Xinjiang People’s Republic of China; 3 Mangistau Regional Multifunctional Hospital, 130000 Aktau City, Mangistau Region Republic of Kazakhstan; 4 Private Hospital Neiron, 130000 Aktau City, Mangistau Region Republic of Kazakhstan; 5 The Second Department of General Surgery, Xinjiang Uygur Autonomous Region Sixth People’s Hospital, Ürümqi, 830000 Xinjiang People’s Republic of China

**Keywords:** Tuberculous spondylitis, Deep learning, Explainable AI (XAI), Convolutional neural network (CNN), MRI sequences

## Abstract

**Background:**

The differentiation of brucellar spondylitis (BS) from tuberculous spondylitis (TS) on magnetic resonance imaging (MRI) is a critical clinical challenge. While deep learning holds promise, the optimal architectural strategy for integrating information from multi-sequence MRI remains unclear. This study systematically compared distinct deep learning architectures to identify a valid and effective integration strategy for this diagnostic problem.

**Methods:**

In this retrospective, single-center diagnostic study, we included 235 patients with surgically and pathologically confirmed BS (*n* = 82) or TS (*n* = 153) from January 2014 to December 2024. We systematically evaluated four distinct architectural strategies for processing sagittal T1-weighted, T2-weighted, and fat-suppressed MRI sequences: (1) baseline models trained on single sequences; (2) a single-branch model that fused sequences as input channels; (3) a heterogeneous multi-branch model using different backbones for each sequence; and (4) a homogeneous multi-branch model using identical backbones. Models were developed on patient-level data splits for training (70%), validation (15%), and internal testing (15%). The primary performance metric was the area under the receiver operating characteristic curve (AUC) on the test set. Statistical significance of performance differences between models was assessed using the DeLong test, with *P* values adjusted for multiple comparisons using the Benjamini–Hochberg procedure.

**Results:**

The single-branch fusion model, which treated the three sequences as channels in a single input, failed to learn, yielding performance equivalent to random chance (test AUC range: 0.474–0.538). In stark contrast, both the single-sequence and multi-branch architectures proved to be effective. The best single-sequence model achieved a test AUC of 0.765 (95% CI 0.759–0.771). The optimal multi-branch model, which successfully integrated all three sequences, achieved a comparable test AUC of 0.764 (95% CI 0.757–0.770).

**Conclusions:**

The choice of architecture for integrating multi-sequence MRI data is a critical determinant of model viability. Our findings demonstrate that naive channel wise fusion is an invalid strategy for this task. In contrast, both processing a single MRI sequence and utilizing a multi-branch parallel-processing architecture are valid and effective strategies, achieving comparable diagnostic performance. This study clarifies the architectural principles required for successfully applying deep learning to this multi-modal diagnostic challenge.

**Supplementary Information:**

The online version contains supplementary material available at 10.1186/s40001-025-03731-9.

## Introduction

Spondylitis, characterized by inflammation of the vertebrae, presents significant clinical challenges, particularly in distinguishing between brucellar spondylitis (BS) and tuberculous spondylitis (TS). Accurate and timely differential diagnosis between BS and TS is crucial due to the severity and potential complications associated with these conditions. Both infections are prevalent in endemic regions, with studies showing either near-equal prevalence or a slightly higher incidence of TS compared to BS [1, 2]. Both conditions share overlapping clinical presentations and can lead to severe complications if misdiagnosed. However, TS is often more severe, associated with a higher rate of spinal complications, such as gibbus deformity, kyphosis, and scoliosis, as well as neurologic deficits, such as loss of sensation, motor weakness, and paralysis. Moreover, TS patients are more likely to require surgical interventions and abscess drainage, emphasizing the need for an accurate diagnosis to guide appropriate and timely treatment [2].

Both BS and TS pose significant health risks and contribute to a substantial economic burden on healthcare systems. The prolonged treatment required for these conditions, often involving long-term antibiotic therapy and potential surgical interventions, leads to increased healthcare costs and absenteeism from work [3]. Current diagnostic methods for spondylitis, including conventional imaging and laboratory tests, often lack the sensitivity and specificity required for reliable differentiation. For BS, blood cultures are considered the gold standard but yield positive results in only 35.5–68.8% of cases, while serological assays such as the standard tube agglutination (STA) test demonstrate higher sensitivity (80.1%) [2]. Imaging, particularly MRI, is highly sensitive for detecting spondylitis, with abnormal vertebral or intervertebral signals observed in 100% of BS cases and disc space narrowing in 88% [4]. However, the microbiological yield is relatively low, with positive culture results in only approximately 33% of pyogenic spondylitis cases and 61% of tuberculous spondylitis cases [5, 6]. CT-guided percutaneous spinal biopsy (CTSB) is a diagnostic procedure commonly employed to assess spinal infections, including BS and TS. Although CTSB is reliable for histopathological diagnosis, its microbiological yield remains limited, which complicates the differentiation between BS and TS based on culture results alone [5, 7, 8]. The similarities in clinical manifestations and imaging findings underscore the need for dependable differential diagnostic criteria. Conventional MRI-based imaging analysis has also some limitations in accurately diagnosing BS and TS due to its inability to differentiate these conditions from other spinal infections with similar radiological features. In addition, MRI may not always detect early stage or subtle changes in the spine, leading to delayed diagnosis and treatment for these diseases [9, 10]. The chronic nature of these diseases and the potential for severe complications underscored their impact on both individual health and broader healthcare systems. Recent advancements in imaging techniques, combined with artificial intelligence (AI) applications, offer a promising avenue to enhance diagnostic accuracy. AI, particularly through deep learning approaches, has the potential to improve the diagnostic process by analyzing complex imaging data that may be challenging for human interpretation alone.

Deep learning architectures, such as convolutional neural networks (CNNs) and transformers, have revolutionized medical imaging by enabling automated feature extraction and classification of imaging data. These models can learn intricate patterns within MRI sequences that may indicate specific diseases. Previous studies have explored the application of AI in differentiating between types of spondylitis, yet significant gaps remain in literature [11]. Many existing models lack thorough validation across diverse patient populations. In addition, they seldom integrate all three imaging sequences—T1-weighted, T2-weighted, and fat-suppressed weighted imaging—into a single network architecture [11]. Furthermore, few models incorporate Explainable AI (XAI) methods to clarify the rationale behind their diagnostic predictions [12, 13]. Insufficient use of all three imaging sequences represents a missed opportunity to maximize medical resources, as each sequence—T1, T2, and FS—provides unique and complementary information for patient evaluation. Integrating the three imaging sequences with XAI techniques is essential for enabling a thorough and transparent decision-making process. This approach enhances the interpretability of AI results, fostering trust and understanding among healthcare professionals.

This study seeks to fill important gaps in the diagnosis of BS and TS by conducting a comprehensive comparative analysis of various deep learning architectures applied to three distinct MRI sequences. By employing a multi-branch network, we aim to leverage the distinct characteristics of each MRI sequence, thereby enhancing the model’s ability to differentiate between BS and TS with greater accuracy. The primary objective is to create a robust diagnostic model that not only distinguishes between these conditions but also integrates XAI techniques, ensuring transparency and interpretability in its decision-making process. This is achieved by fully utilizing the information from each MRI sequence within the network architecture, thus enhancing diagnostic precision and the overall performance of the model. This study made several key contributions. The main contributions of this study are: (1) a systematic, comparative analysis of distinct deep learning architectures—single-sequence, single-branch fusion, and multi-branch-for differentiating BS and TS; (2) the establishment of a critical architectural principle by demonstrating that naive channelwise fusion is an invalid strategy, whereas parallel processing (via either a single-sequence or a multi-branch model) represents a valid and effective approach; and (3) the integration of explainable AI (XAI) techniques to provide transparent insight into model decision-making, a crucial step for building clinical trust.

## Methods

This retrospective study was approved by the Ethics Committees of The Sixth Affiliated Hospital of Xinjiang Medical University. Given the retrospective nature of the study and the de-identification of the data, the requirement for individual agreements and written informed consent was waived.

### Population and study design

**Fig. 1:**
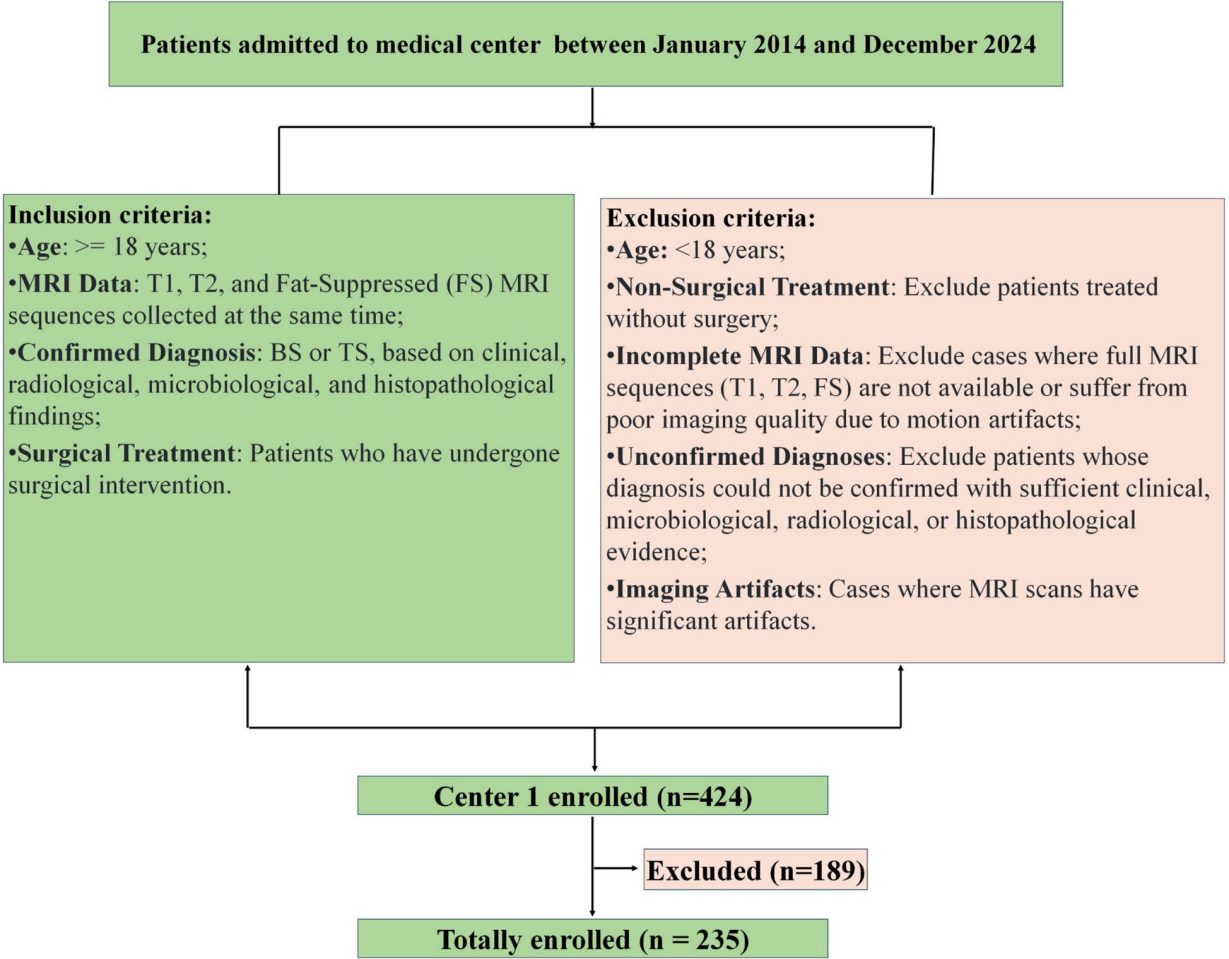
Population recruitment workflow of this research

This study examined a data set of 235 patients who underwent surgical treatment for spinal infections at our medical center between January 2014 and December 2024. The data set was split into training (70%), validation (15%), and testing (15%) subsets using stratified sampling based on diagnosis to preserve the class distribution (BS:TS ≈ 1:1.87). The data were sourced from The Sixth Affiliated Hospital of Xinjiang Medical University. A retrospective design was employed to evaluate the performance of various deep learning models in analyzing MRI sequences (T1, T2, and FS) to differentiate between tuberculous spondylitis (TS) and bacterial spondylitis (BS). Patient selection focused on individuals who had undergone MRI scans with corresponding clinical data available. The inclusion criteria ensured a representative sample, while exclusion criteria removed cases with incomplete data or potential confounders (see Fig. 1).

The inclusion criteria were as follows: (1) patients aged 18 years or older were included to ensure adult representation. (2) Only patients who had undergone surgical treatment for spinal infections, such as decompression, fusion, or abscess drainage, were eligible. (3) Patients who had MRI scans (T1, T2, and FS sequences) at the meantime as part of their diagnostic workup were considered. (4) Complete clinical records, including demographic data, medical history, and laboratory results, were required for a comprehensive evaluation. (5) Patients with a confirmed diagnosis of either TS or BS, based on clinical, radiological, microbiological, and histopathological findings, were included [14]. Patients were excluded if they received only non-surgical treatment, had incomplete or poor-quality MRI sequences, lacked comprehensive clinical documentation, or had inconclusive diagnoses that could not be definitively classified as either TS or BS.

### MR examinations

This study utilized 1.5 Tesla MR scanners from various manufacturers. Multiple imaging sequences were employed, including sagittal T1-weighted images (T1WI), axial and sagittal T2-weighted images (T2WI), and sagittal fat-suppressed T2-weighted images (FS-T2WI). Detailed imaging parameters for each sequence are provided in Supplementary Table 1. We acquired and stored images formatted according to Digital Imaging and Communications in Medicine (DICOM) standards using the Picture Archiving and Communication System (PACS).

### ROI segmentation, extraction and augmentation

Two experienced spine surgeons, with 5 (J. S.) and 20 (X.H.S.) years of expertise in spinal infectious imaging, independently reviewed and contoured all sagittal MR slices, including T1, T2, and FS sequences. Both were blinded to clinical details, laboratory test results, and histopathological findings. The annotation was conducted using the open-source software platform 3D Slicer (version 5.6.2; https://www.slicer.org). The extracted regions of interest (ROIs) underwent thorough preprocessing to ensure consistency and reliability for subsequent analyses. A custom computational framework was implemented to convert the segmentation masks from their original nrrd format into standardized images (png), ensuring compatibility with machine learning workflows. Each ROI was isolated using a bounding box approach, specifically targeting the infected spinal regions while excluding irrelevant anatomical structures. To mitigate variations in MR imaging protocols, intensity normalization was applied across all sequences, standardizing pixel values to a consistent scale between 0 and 255. This step aimed to reduce inter-scan variability and enhance feature comparability across data sets. In addition, spatial alignment of the MR images and segmentation masks was achieved using linear interpolation, ensuring uniform image dimensions while preserving anatomical accuracy. The T1-weighted sequence was designated as the fixed image to ensure anatomical fidelity during linear interpolation and alignment of the MR images and segmentation masks. Following these preprocessing steps, we obtained 185,178 images for training, validation and testing data sets. Original image dimensions ranged from 512 × 512 to 1024 × 1024 pixels, depending on the sequence; all were resized and interpolated to 224 × 224 pixels using bilinear interpolation for model input consistency.

The annotated and preprocessed ROIs underwent an extensive data augmentation process to enhance data set diversity and improve the robustness of subsequent machine learning analyses. A custom Python framework, utilizing OpenCV, was employed to systematically apply a series of geometric transformations. These included rotations at 12 predefined angles (0°, 30°, 60°, 90°, 120°, 150°, 180°, 210°, 240°, 270°, 300°, and 330°) and flips along both the horizontal and vertical axes. These transformations were applied to both the segmentation masks and corresponding MR images to preserve spatial consistency and maintain the integrity of anatomical features. Augmentation was performed after ROI extraction. The images were rotated and flipped within their bounding boxes. We have now clarified that any empty space created by rotation was filled with black pixels (value 0). The augmented images and masks were saved in a standardized format, with filenames encoding transformation parameters, ensuring traceability and reproducibility. This augmentation pipeline addressed potential class imbalances and reduced the risk of overfitting by exposing machine learning models to a wider range of spatial variations, thus facilitating the development of generalizable and reliable predictive algorithms for spinal infection diagnosis. To prevent data leakage, patient-level stratification was maintained during data set splitting, ensuring all images from an individual patient remained in the same subset.

### Model development

The development of the proposed models for classifying MRI sequences (T1, T2, FS) into TS and BS included a progressive pipeline of four distinct approaches, each designed to address specific challenges and improve classification accuracy. This approach aims to leverage a single pre-trained model to process multiple MRI sequences, facilitating a comparison of different architectures. All model architectures, input sizes, and initial weights are provided in Supplementary Table 2.

#### Baseline model

**Fig. 2:**
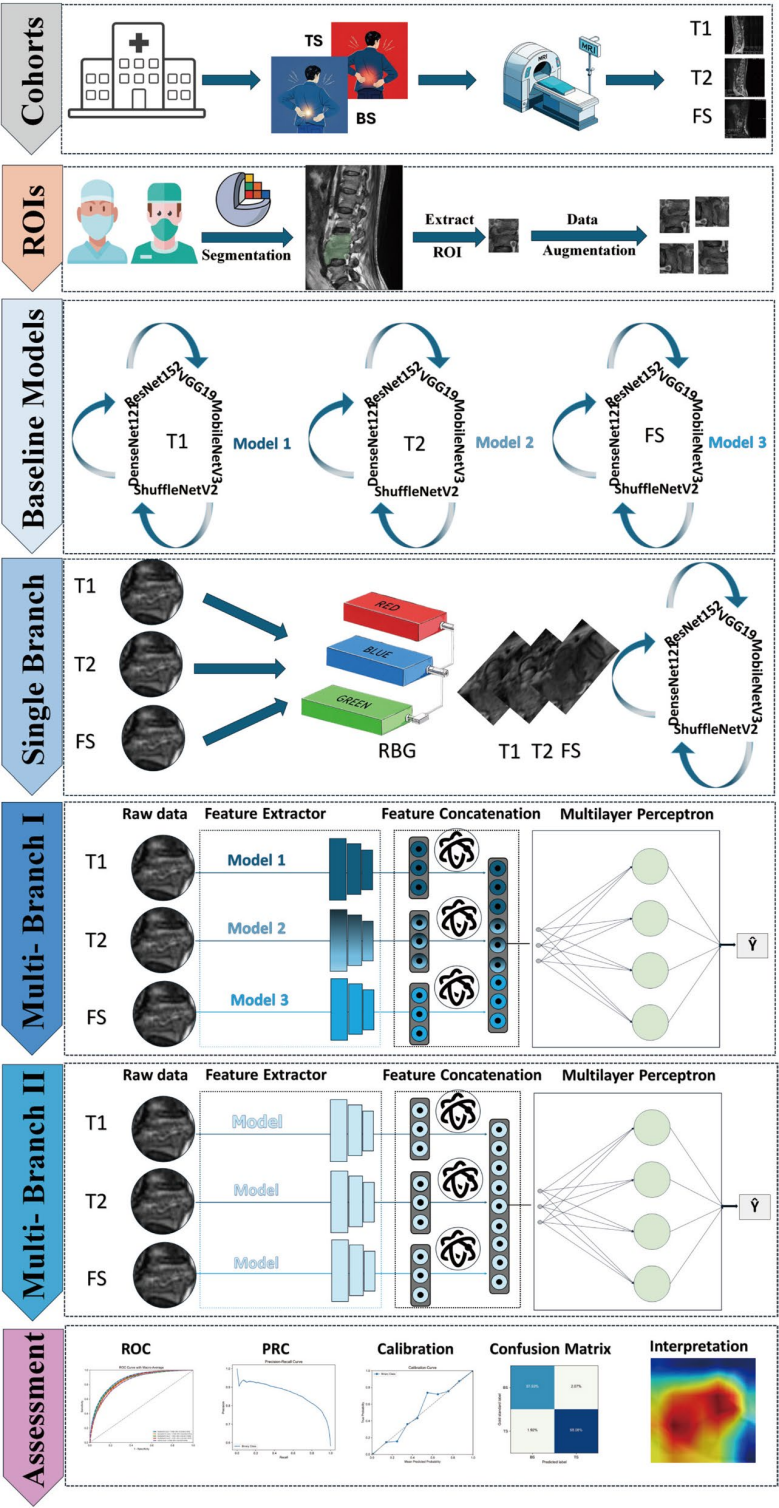
Flowchart of whole study

The primary goal of this approach is to independently train each MRI sequence (T1, T2, FS) using different networks, establishing a baseline for comparison. The MRI sequences—T1, T2, and FS—serve as the input data for this process. Each network, including ResNet152, DenseNet121, VGG19, MobileNetV3, and ShuffleNetV2 was trained on one of the MRI sequences. The performance of each model is then recorded and evaluated. The output of this approach consists of performance metrics for each model. This framework acts as a baseline for identifying the best-performing network for each MRI sequence, which will inform the selection of models for subsequent stages of the study (see Fig. 2). The detailed training process and parameters are available in **Appendix S1**.

#### Single-branch model development

To address the challenge of integrating multiple MRI sequences (T1, T2, and FS), a single-branch model was implemented. This model used a multi-channel input structure, where the T1, T2, and FS images for each patient were stacked along the channel dimension to form a three-channel representation. A custom data set class was developed to manage data loading and preprocessing, ensuring the proper alignment of the T1, T2, and FS images for each case. All images were resized to a consistent spatial resolution of 224 × 224 pixels, and various augmentation techniques, such as rotation, flipping, zooming, and cropping, were applied during training to enhance generalization. Augmentation was applied post-ROI extraction in preprocessing to generate a diverse data set, and additional on-the-fly augmentations were used during training to enhance robustness, with both steps optimized to avoid overlap and ensure efficient resource use (see Fig. 2).

The three-channel input was then processed through deep learning architectures, including DenseNet121, ResNet152, MobileNetV3, ShuffleNetV2, and VGG19. These models were adapted to accept three-channel inputs by modifying their first convolutional layers. The final classification layer of each model was replaced with a fully connected layer to enable binary prediction. This single-branch approach enabled the models to learn a unified representation of the three MRI sequences, effectively capturing complementary information for accurate classification. Details of the training process and parameters can be found in Appendix S2.

#### Multi-branch model development

The proposed multi-branch model was developed to integrate multi-sequence MRI images (T1, T2, and FS) into a unified classification network. The architecture consisted of three parallel branches—each dedicated to extracting features from one MRI sequence—followed by a shared fully connected layer for final classification. The composition of this heterogeneous multi-branch model was determined by a two-stage sequential process. First, we conducted the baseline single-sequence experiments, as described in "Baseline model" section. Second, for each of the three MRI sequences (T1, T2, and FS), we identified the backbone architecture that demonstrated the best generalization performance on the internal test set. These three selected backbones were then assembled as the parallel branches of the final heterogeneous model, whose performance was subsequently evaluated. The outputs of the three branches were flattened and concatenated to form a combined feature vector, which was then passed through a fully connected layer to classify the images into outcome (see Fig. 2). This approach allowed the network to leverage unique features from each MRI sequence while maintaining a shared decision-making process. The model design effectively addressed the challenge of combining multi-sequence data into a single framework, enabling robust and accurate classification of spinal pathologies. Details of the training process and parameters can be found in Appendix S3.

#### Comparative multi-branch model development

The comparative multi-branch model was designed to evaluate the performance of a unified network architecture that integrates three distinct MRI sequences (T1, T2, and FS). It consisted of three parallel branches, each dedicated to one modality, allowing for the extraction of complementary features. Each branch utilized the same backbone model (e.g., DenseNet121, ResNet152, MobileNetV3, ShuffleNetV2, and VGG19), pre-trained on ImageNet, to ensure consistent feature extraction across modalities. The outputs of the branches were flattened, concatenated, and passed through a fully connected layer for classification (see Fig. 2). This architecture allowed the model to capture modality-specific information while also leveraging shared learning for improved classification, creating a robust framework for integrating multi-sequence data. By standardizing the branch architecture, the model facilitated a direct comparison of how different backbone architectures affected classification performance, ensuring both reproducibility and interpretability. Further details on the training process and parameters are provided in Appendix S4.

### Model explanation

The model explanation section elucidates the interpretability of the proposed network through advanced visualization techniques. By employing various Class Activation Mapping (CAM) methods, such as GradCAM, HiResCAM, and LayerCAM, we provide insights into the decision-making process of the final convolutional layer of each model [15]. These techniques highlight the regions of the input images that contribute most significantly to the model's predictions, thereby enhancing the transparency and trustworthiness of the model. The CAM visualizations are generated for each positive class label, allowing for a detailed examination of the model's focus different outcomes.

### Statistical analysis

The performance of the baseline models, single-branch model, and multi-branch model were rigorously evaluated using validation, and testing data sets. Statistical analyses were conducted to compare categorical variables using the chi-square test or Fisher's exact test, with results expressed as frequencies and percentages. Continuous variables following a normal distribution were summarized as mean ± standard deviation (SD) and compared using a two-tailed Student's *t* test. To ensure robust and comprehensive performance evaluation, the following metrics were computed for each class: accuracy, sensitivity, specificity, precision (positive predictive value, PPV), negative predictive value (NPV), F1-Score, Matthews correlation coefficient (MCC), false negative rate (FNR), false positive rate (FPR), false discovery rate (FDR), and the area under the receiver operating characteristic curve (AUC) with associated 95% confidence intervals (AUC 95% CI). These metrics were calculated using Python 3.9.10 with scikit-learn, ensuring reproducibility. In addition, graphical representations, including Receiver Operating Characteristic (ROC) curves and calibration plots, were generated using R Studio 4.4.0 and Python, providing clear visualizations of model performance and statistical comparisons. Statistical differences in AUC between models were assessed with DeLong's test, and *P* values were adjusted for multiple testing via the Benjamini–Hochberg method [16, 17]. Statistical significance was defined as a two-sided *P* value < 0.05.

## Results

### Patients characteristics

The study analyzed a total of 235 participants, stratified into two groups: TS (*n* = 153) and BS (*n* = 82). Demographic characteristics revealed a mean age of 47.1 ± 20.0 years across all participants, with no significant differences between the groups. The cohort consisted of 144 females (61.3%) and 91 males (38.7%), with a higher proportion of females in the TS group (64.7%) compared to the BS group (54.9%) (see Supplementary File Table 3).

### Baseline model performance

**Fig. 3:**
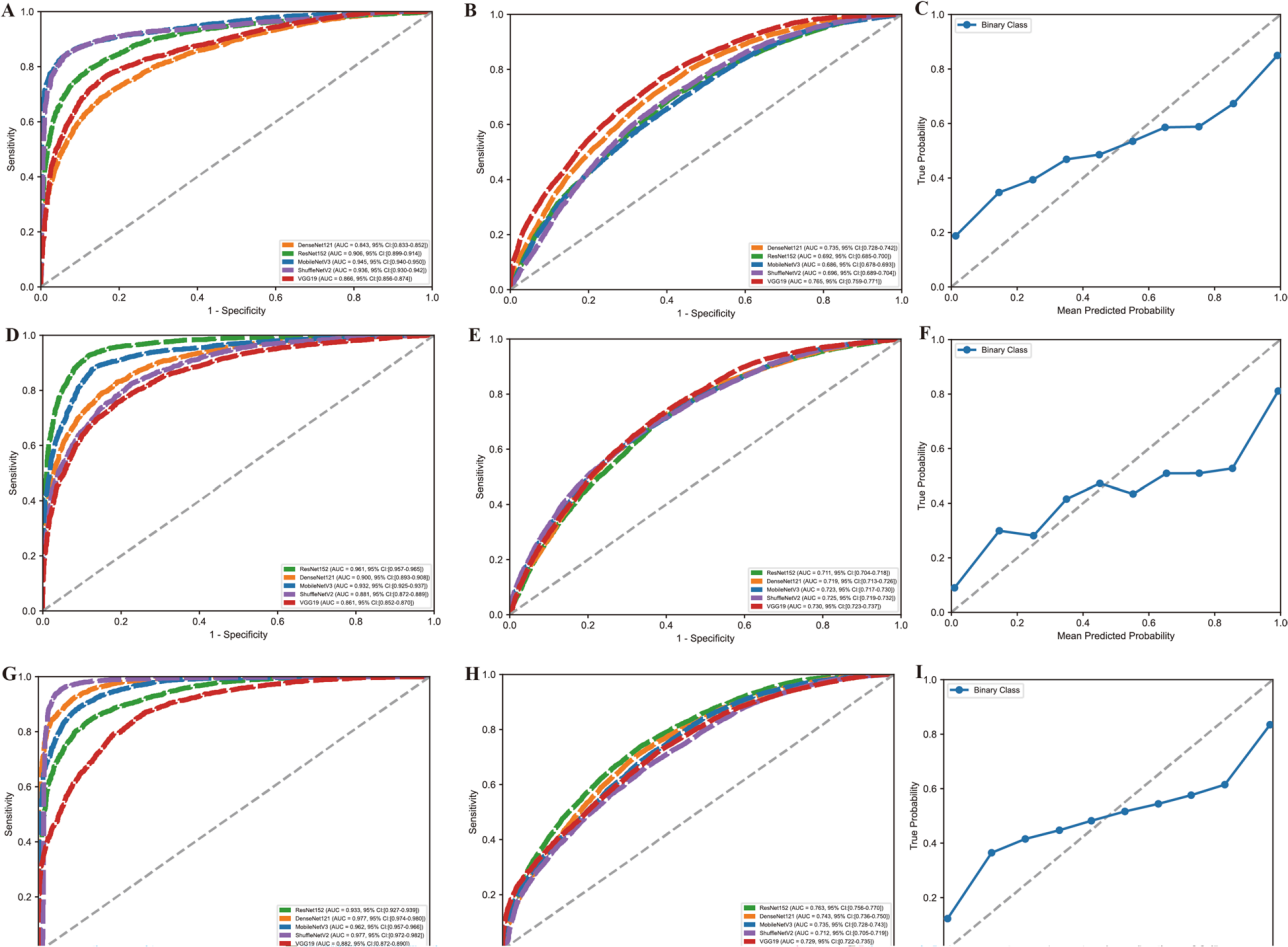
Model performance across data sequences. **A** Receiver operating characteristic (ROC) curves for the T1-weighted validation set, comparing the performance of different models (e.g., ResNet152, DenseNet121, MobileNetV3, ShuffleNetV2, and VGG19) in distinguishing between Brucellar Spondylitis (BS) and Tuberculous Spondylitis (TS); **B**: ROC curves for the T1-weighted testing set, evaluating model generalization on unseen data; **C** calibration plot for the T1-weighted testing set, specifically for the VGG19 model, showing the alignment between predicted probabilities and actual outcomes; **D** ROC curves for the T2-weighted validation set, comparing model performance across architectures; **E** ROC curves for the T2-weighted testing set, assessing generalization ability; **F** calibration plot for the T2-weighted testing set, specifically for the ShuffleNetV2 model, illustrating the relationship between predicted and true probabilities; **G** ROC curves for the fat-suppressed (FS) validation set, evaluating model discrimination; **H** ROC curves for the FS testing set, demonstrating performance on unseen data; **I** calibration plot for the FS testing set, specifically for the ResNet152 model, highlighting calibration accuracy

The baseline models, trained independently on single MRI sequences, demonstrated a consistent pattern of high performance on the validation set followed by a notable performance decrease on the internal test set, indicating a degree of overfitting across all architectures and sequences (Fig. 3). For models trained on T1-weighted images, MobileNetV3 achieved the highest area under the receiver operating characteristic curve (AUC) of 0.945 (95% CI 0.940–0.950) on the validation data (see Fig. 3A). On the test set, however, all models showed reduced efficacy, with VGG19 yielding the best performance at an AUC of 0.765 (95% CI 0.759–0.771) (see Fig. 3B). This trend was mirrored in the T2-weighted sequence evaluation, where ResNet152 was the top performer on the validation set (AUC = 0.961, 95% CI 0.957–0.965) (see Fig. 3D). Nevertheless, VGG19 and ShuffleNetV2 performed best on the test set with AUCs of 0.730 (95% CI 0.723–0.737) and 0.725 (95% CI 0.719–0.732) (see Fig. 3E). Models trained on fat-suppressed (FS) sequences achieved the highest validation performance, with ShuffleNetV2 attaining a near-perfect AUC of 0.977 (95% CI 0.974–0.980) (see Fig. 3G). On the test set, this performance also declined, with ResNet152 providing the best discrimination with an AUC of 0.763 (95% CI 0.756–0.770) (see Fig. 3H). Based on their superior generalization to the test set, VGG19 (T1-weighted), ShuffleNetV2 (T2-weighted), and ResNet152 (fat-suppressed) were selected; however, calibration plots for all three revealed imperfect alignment between predicted probabilities and true outcomes, as their curves consistently deviated from the line of perfect calibration on testing set (see Fig. 3C, F, I and Table 1).

**Table 1:** Comprehensive performance assessment of all evaluated deep learning architectural strategies on validation and testing sets

Model type	Model name	ACC	F1S	FDR	FNR	MCC	NPV	PRE	REC
				Validation set					
Single sequence	T1-VGG19	0.732	0.805	0.311	0.312	0.376	0.689	0.801	0.809
Single sequence	T2-ShuffleNetV2	0.680	0.755	0.356	0.342	0.302	0.645	0.797	0.717
Single sequence	FS-ResNet152	0.738	0.815	0.306	0.324	0.37	0.694	0.79	0.841
Single branch (fusion)	SingleBranch-ResNet152	0.684	0.812	0.158	0.500	0	0.342	0.684	1.000
Heterogeneous multi-branch	Hetero-VSR	0.913	0.926	0.094	0.082	0.824	0.906	0.963	0.891
Homogeneous multi-branch	Homo-MobileNetV3	0.922	0.932	0.084	0.066	0.849	0.916	0.992	0.879
Homogeneous multi-branch	Homo-DenseNet121	0.932	0.942	0.075	0.062	0.863	0.925	0.976	0.911
Homogeneous multi-branch	Homo-ResNet152	0.821	0.841	0.182	0.166	0.651	0.818	0.917	0.777
Homogeneous multi-branch	Homo-VGG19	0.824	0.858	0.184	0.188	0.628	0.816	0.846	0.87
Homogeneous multi-branch	Homo-ShuffleNetV2	0.866	0.886	0.143	0.133	0.724	0.858	0.914	0.861
				Testing set					
Single sequence	T1-VGG19	0.732	0.805	0.311	0.312	0.376	0.689	0.801	0.809
Single sequence	T2-ShuffleNetV2	0.680	0.755	0.356	0.342	0.302	0.645	0.797	0.717
Single sequence	FS-ResNet152	0.738	0.815	0.306	0.324	0.370	0.694	0.790	0.841
Single branch (fusion)	SingleBranch-ResNet152	0.684	0.812	0.158	0.500	0.000	0.342	0.684	1.000
Heterogeneous Multi-Branch	Hetero-VSR	0.718	0.792	0.325	0.322	0.353	0.675	0.798	0.786
Homogeneous Multi-Branch	Homo-MobileNetV3	0.660	0.726	0.360	0.341	0.299	0.640	0.807	0.660
Homogeneous Multi-Branch	Homo-DenseNet121	0.699	0.774	0.342	0.334	0.324	0.658	0.794	0.755
Homogeneous Multi-Branch	Homo-ResNet152	0.653	0.726	0.374	0.358	0.268	0.626	0.789	0.673
Homogeneous Multi-Branch	Homo-VGG19	0.715	0.802	0.336	0.360	0.302	0.664	0.763	0.846
Homogeneous Multi-Branch	Homo-ShuffleNetV2	0.717	0.789	0.324	0.316	0.360	0.676	0.804	0.774

Model Type: Single-Branch Fusion, models fusing features into a single channel; Heterogeneous Multi-Branch, strategy utilizing different backbones for different MRI sequences; Homogeneous Multi-Branch, strategy utilizing identical backbones for all sequences. Model Names: T1-VGG19, VGG19 trained on T1 sequences; Hetero-VSR, Heterogeneous model combining VGG19 (T1), ShuffleNetV2 (T2), and ResNet152 (FS); Homo-Model Name (e.g., Homo-ShuffleNetV2), Homogeneous multi-branch model using the specified backbone for all three sequences

ACC, Accuracy; AUC, Area Under the Curve; CI, Confidence Interval; F1S, F1-Score; MCC, Matthews Correlation Coefficient; NPV, Negative Predictive Value; PRE, Precision; REC, Recall; Spec., Specificity

### Single-branch model performance

We implemented a single-branch model utilizing a CNN architecture to differentiate between BS and TS by processing MRI sequences as three-channel RGB inputs, where each channel corresponded to T1, T2, and FS imaging characteristics. On the validation set, the models demonstrated limited discriminatory ability, with ROC curves clustering near the diagonal line of no-discrimination (see Supplementary Fig. 1A). ResNet152 achieved the highest area under the curve (AUC) of 0.534 (95% CI 0.520–0.548), followed by ShuffleNetV2 (AUC = 0.503, 95% CI 0.487–0.518) and VGG19 (AUC = 0.505, 95% CI 0.500–0.510). DenseNet121 and MobileNetV3 performed poorest, with AUCs of 0.493 (95% CI 0.479–0.509) and 0.445 (95% CI 0.430–0.459), respectively. This performance trend was largely consistent on the internal testing set (see Supplementary Fig. 1B), where ShuffleNetV2 and ResNet152 again showed slightly better, albeit still poor, performance with AUCs of 0.534 (95% CI 0.526–0.541) and 0.538 (95% CI 0.530–0.546). The other models, including VGG19 (AUC = 0.500, 95% CI 0.497–0.503), DenseNet121 (AUC = 0.486, 95% CI 0.478–0.494), and MobileNetV3 (AUC = 0.474, 95% CI 0.465–0.481), all performed near chance level, indicating that the single-branch architectural approach struggled to effectively differentiate between brucellar and tuberculous spondylitis using the combined MRI sequences (see Table 1).

### Multi-branch model performance

**Fig. 4:**
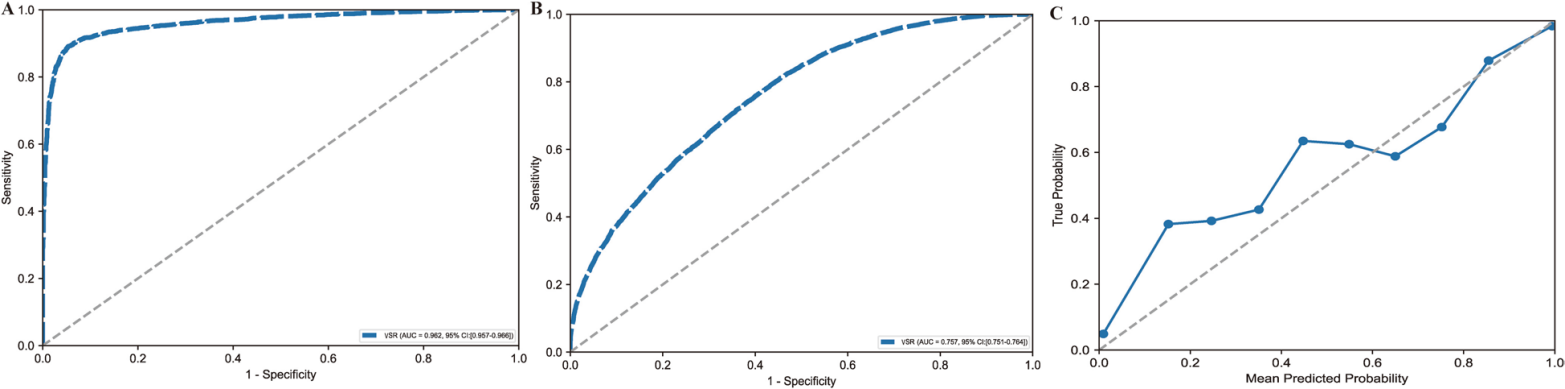
Multibranch model performance. This model was constructed by integrating the best-performing single-sequence backbones: VGG19 (T1), ShuffleNetV2 (T2), and ResNet152 (FS). **A** Receiver operating characteristic (ROC) curves and corresponding area under the curve (AUC) values for the model's performance on the validation set. **B** Final performance of the model shown by the ROC curve and AUC on the unseen testing set. **C** Calibration plot for the model on the testing set, evaluating the reliability of its predicted probabilities against the observed outcomes

Specifically, based on the results of the baseline experiments ("Baseline model performance" section), we constructed a heterogeneous multi-branch model (denoted as hetero-VSR in Table 1). This architecture integrated VGG19 for the T1-weighted branch, ShuffleNetV2 for the T2-weighted branch, and ResNet152 for the FS branch. Figure 4A illustrates the ROC curve for the validation set, achieving an AUC of 0.962 (95% CI 0.957–0.966), which demonstrated strong discriminative capability. Figure 4B depicts the ROC curve for the testing set, where the AUC decreased to 0.757 (95% CI 0.751–0.764), indicating a decline in generalization performance. Figure 4C presents a calibration plot for the testing set, revealing a close alignment between predicted probabilities and true outcomes, which underscored the model's reliability in predictive confidence. Collectively, these findings emphasized the hetero-VSR model's high accuracy on validation data but highlighted challenges in maintaining performance on unseen data.

**Fig. 5:**
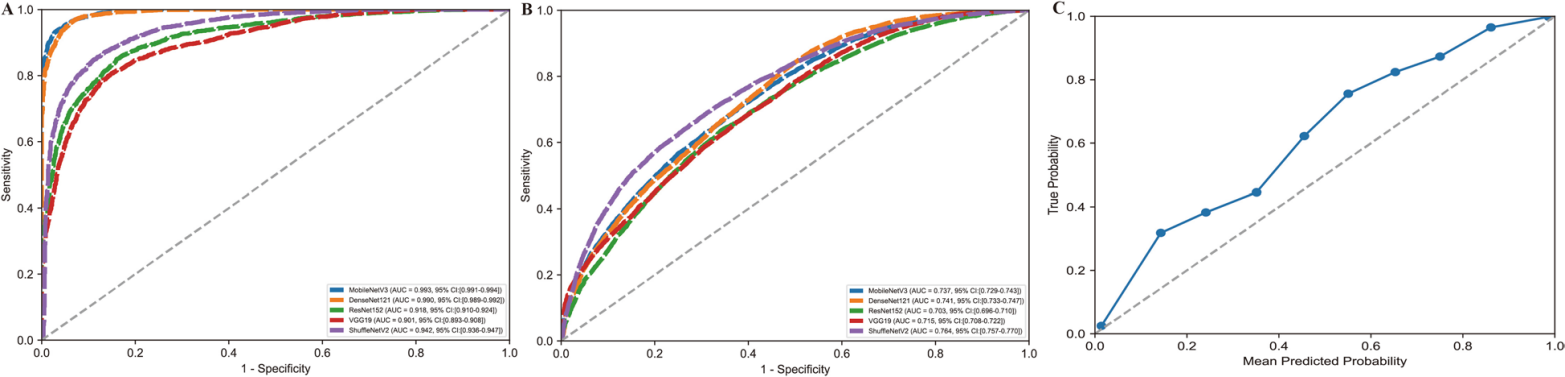
Multibranch network performance. This figure compares five models, where each model uses the same backbone architecture (ResNet152, DenseNet121, MobileNetV3, ShuffleNetV2, or VGG19) for all three parallel branches. **A** ROC curves and AUC values comparing the performance of the five distinct models on the validation set. **B** ROC curves and AUC values for the same five models on the testing set, evaluating their generalization performance. **C** Calibration plot for the best-performing homogeneous model (ShuffleNetV2) on the testing set, illustrating the alignment between its predicted probabilities and actual frequencies

We further implemented a homogeneous multi-branch strategy (see Table 1) to concurrently process multiple data types. Unlike the heterogeneous approach, this strategy duplicated a single backbone architecture to create three identical parallel branches for simultaneously analyzing T1, T2, and FS data. For instance, Homo-DenseNet121 utilized three identical DenseNet121 networks. Figure 5A displays the ROC curves for the validation set, where Homo-MobileNetV3 achieved the highest AUC of 0.993 (95% CI 0.991–0.994), followed by Homo-DenseNet121 (AUC = 0.990, 95% CI 0.989–0.992). Figure 5B depicts the ROC curves for the testing set, with Homo-ShuffleNetV2 attaining the highest AUC of 0.764 (95% CI 0.757–0.770), outperforming other homogeneous models, such as Homo-MobileNetV3 (AUC = 0.737) and Homo-DenseNet121 (AUC = 0.741). Figure 5C presents the calibration plot for the best-performing model, Homo-ShuffleNetV2, on the testing set, providing a detailed assessment of the agreement between predicted probabilities and observed outcomes. Further evaluation of Homo-ShuffleNetV2 and related results are provided in Supplementary Fig. 2.

To statistically validate our architectural insights, we performed a comprehensive pairwise comparison of all models (Table 2). The analysis yielded two key findings that support our architectural hypothesis. First, the single-branch fusion strategy (e.g., SingleBranch–ResNet152) was statistically inferior to all valid strategies, demonstrating significantly lower AUCs than both the best single-sequence model (T1-VGG19, adjusted *P* < 0.001) and the best multi-branch model (Homo-ShuffleNetV2, adjusted *P* < 0.001). Second, and most importantly, no statistically significant difference was observed between the optimal single-sequence approach (T1-VGG19) and the optimal multi-branch approach (Homo-ShuffleNetV2), with an AUC difference of only 0.001 and an adjusted *P* value of 0.861. This statistical evidence confirms that while the homogeneous multi-branch architecture is a valid integration strategy, it does not strictly outperform a well-optimized single-sequence model for this specific diagnostic task.

**Table 2:** Pairwise statistical comparison of AUCs between architectural strategies using DeLong's test with Benjamini–Hochberg correction

Model1	Model2	AUC1	AUC2	AUC Difference	*P* value	Adjusted *P* value
T1-VGG19	SingleBranch-ResNet152	0.765	0.538	0.227	< 0.001	< 0.001
FS-ResNet152	SingleBranch-ResNet152	0.763	0.538	0.225	< 0.001	< 0.001
SingleBranch-ResNet152	Hetero-VSR	0.538	0.757	− 0.219	< 0.001	< 0.001
SingleBranch-ResNet152	Homo-ShuffleNetV2	0.538	0.764	− 0.226	< 0.001	< 0.001
SingleBranch-ResNet152	Homo-DenseNet121	0.538	0.741	− 0.202	< 0.001	< 0.001
SingleBranch-ResNet152	Homo-MobileNetV3	0.538	0.737	− 0.199	< 0.001	< 0.001
T2-ShuffleNetV2	SingleBranch-ResNet152	0.725	0.538	0.187	< 0.001	< 0.001
SingleBranch-ResNet152	Homo-VGG19	0.538	0.715	− 0.177	< 0.001	< 0.001
SingleBranch-ResNet152	Homo-ResNet152	0.538	0.703	− 0.165	< 0.001	< 0.001
Homo-ShuffleNetV2	Homo-ResNet152	0.764	0.703	0.061	< 0.001	< 0.001
Homo-ShuffleNetV2	Homo-VGG19	0.764	0.715	0.049	< 0.001	< 0.001
T1-VGG19	Homo-ResNet152	0.765	0.703	0.062	< 0.001	< 0.001
Homo-DenseNet121	Homo-ResNet152	0.741	0.703	0.037	< 0.001	< 0.001
FS-ResNet152	Homo-ResNet152	0.763	0.703	0.060	< 0.001	< 0.001
Hetero-VSR	Homo-ResNet152	0.757	0.703	0.054	< 0.001	< 0.001
Homo-MobileNetV3	Homo-ResNet152	0.737	0.703	0.034	< 0.001	< 0.001
T1-VGG19	Homo-VGG19	0.765	0.715	0.050	< 0.001	< 0.001
FS-ResNet152	Homo-VGG19	0.763	0.715	0.048	< 0.001	< 0.001
Homo-ShuffleNetV2	Homo-MobileNetV3	0.764	0.737	0.027	< 0.001	< 0.001
Hetero-VSR	Homo-VGG19	0.757	0.715	0.043	< 0.001	< 0.001
Homo-DenseNet121	Homo-VGG19	0.741	0.715	0.026	< 0.001	< 0.001
T1-VGG19	T2-ShuffleNetV2	0.765	0.725	0.040	< 0.001	< 0.001
T2-ShuffleNetV2	Homo-ShuffleNetV2	0.725	0.764	− 0.039	< 0.001	< 0.001
Homo-ShuffleNetV2	Homo-DenseNet121	0.764	0.741	0.023	< 0.001	< 0.001
T2-ShuffleNetV2	FS-ResNet152	0.725	0.763	− 0.038	< 0.001	< 0.001
Homo-MobileNetV3	Homo-VGG19	0.737	0.715	0.022	< 0.001	< 0.001
T2-ShuffleNetV2	Hetero-VSR	0.725	0.757	− 0.032	< 0.001	< 0.001
T1-VGG19	Homo-MobileNetV3	0.765	0.737	0.028	< 0.001	< 0.001
FS-ResNet152	Homo-MobileNetV3	0.763	0.737	0.027	< 0.001	< 0.001
T1-VGG19	Homo-DenseNet121	0.765	0.741	0.024	< 0.001	< 0.001
FS-ResNet152	Homo-DenseNet121	0.763	0.741	0.023	< 0.001	< 0.001
T2-ShuffleNetV2	Homo-ResNet152	0.725	0.703	0.022	< 0.001	< 0.001
Hetero-VSR	Homo-MobileNetV3	0.757	0.737	0.021	< 0.001	< 0.001
Homo-ResNet152	Homo-VGG19	0.703	0.715	− 0.012	< 0.001	< 0.001
Hetero-VSR	Homo-DenseNet121	0.757	0.741	0.017	0.001	0.001
T2-ShuffleNetV2	Homo-DenseNet121	0.725	0.741	− 0.015	0.002	0.002
T2-ShuffleNetV2	Homo-MobileNetV3	0.725	0.737	− 0.012	0.020	0.024
T2-ShuffleNetV2	Homo-VGG19	0.725	0.715	0.010	0.040	0.047
T1-VGG19	Hetero-VSR	0.765	0.757	0.007	0.126	0.146
Homo-MobileNetV3	Homo-DenseNet121	0.737	0.741	− 0.004	0.145	0.163
Hetero-VSR	Homo-ShuffleNetV2	0.757	0.764	− 0.006	0.183	0.201
FS-ResNet152	Hetero-VSR	0.763	0.757	0.006	0.218	0.234
T1-VGG19	FS-ResNet152	0.765	0.763	0.001	0.772	0.808
T1-VGG19	Homo-ShuffleNetV2	0.765	0.764	0.001	0.842	0.861
FS-ResNet152	Homo-ShuffleNetV2	0.763	0.764	0.000	0.927	0.927

AUC: Area under the receiver operating characteristic curve. AUC difference = *AUC*Model1—*AUC* Model2

Statistical significance: *P* values were adjusted for multiple comparisons (45 pairs) using the Benjamini–Hochberg (BH) procedure

Model Type: Single-Branch Fusion, models fusing features into a single channel; Heterogeneous Multi-Branch, strategy utilizing different backbones for different MRI sequences; Homogeneous Multi-Branch, strategy utilizing identical backbones for all sequences. Model Names: T1-VGG19, VGG19 trained on T1 sequences; Hetero-VSR, Heterogeneous model combining VGG19 (T1), ShuffleNetV2 (T2), and ResNet152 (FS); Homo-Model Name (e.g., Homo-ShuffleNetV2), Homogeneous multi-branch model using the specified backbone for all three sequences

### Model interpretation

**Fig. 6:**
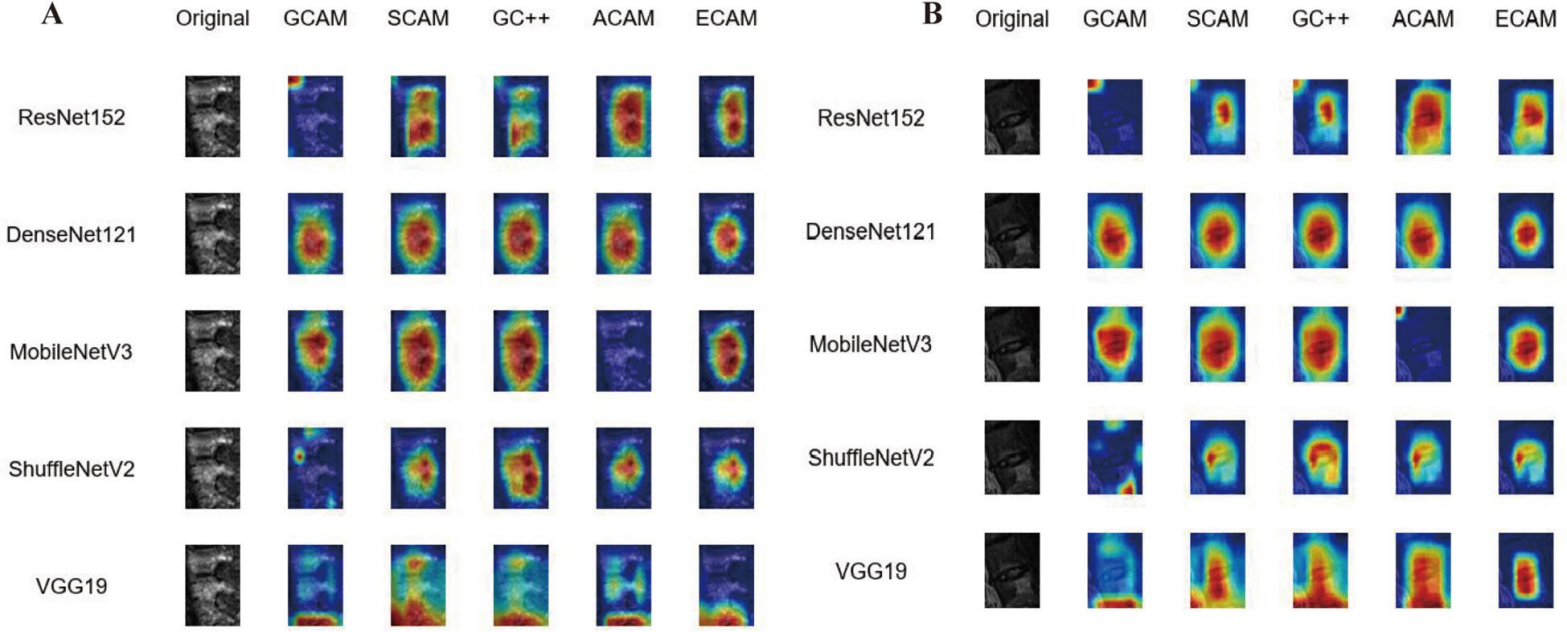
Grad-CAM visualization for single-sequence models. **A** Original images of TS patient and Grad-CAM heatmaps (GCAM: GradCAM, SCAM: ScoreCAM, GC++: GradCAMPlusPlus, ACAM: AblationCAM, and ECAM: EigenCAM) for ResNet152, DenseNet121, MobileNetV3, ShuffleNetV2, and VGG19, showing model attention regions; **B** original images of BS patient and Grad-CAM heatmaps (GCAM: GradCAM, SCAM: ScoreCAM, GC++: GradCAMPlusPlus, ACAM: AblationCAM, and ECAM: EigenCAM) for ResNet152, DenseNet121, MobileNetV3, ShuffleNetV2, and VGG19, showing model attention regions

To understand the basis for the models' predictions, we applied several Class Activation Mapping (CAM) methods to the single-sequence baseline models. We focused this analysis on comparing how the different backbone architectures, when trained on the same data, learned to identify pathological features. These heatmaps highlighted distinct attention regions, revealing differences in activation patterns across architectures. Figure 6A presents the original input images along with the Grad-CAM heatmaps generated by five different methods (GCAM: GradCAM, SCAM: ScoreCAM, GC++: GradCAMPlusPlus, ACAM: AblationCAM, and ECAM: EigenCAM) for each model, effectively highlighting regions of interest in the input data. Figure 6B repeats this analysis with a different image, revealing consistent activation patterns across the architecture. These visualizations underscored how the various Grad-CAM variants emphasized distinct spatial regions, offering valuable insights into model interpretability and feature importance. The heatmaps displayed notable differences in both activation intensity and localization, showcasing how each model processed the input features to inform its predictions.

## Discussion

BS and TS were significant infectious diseases that affected the spine, leading to chronic pain, disability, and substantial healthcare costs. These conditions imposed a considerable economic burden on patients and society due to their morbidity and the challenges associated with their diagnosis and treatment. Current diagnostic methods, such as MRI, and treatments like antibiotic therapy, had limitations, including the risk of misdiagnosis and delayed treatment. This study conducted a comprehensive evaluation of deep learning architectures for differentiating BS and TS through MRI sequences and found variations in model performance across architectures and integration strategies. The baseline models trained on single MRI sequences showed promising but variable performance, with the best testing set performance achieved by VGG19 on T1-weighted images (AUC: 0.765), ShuffleNetV2 on T2-weighted images (AUC: 0.725), and ResNet152 on fat-suppressed sequences (AUC: 0.763). Notably, the single-branch model, which fused all three MRI sequences as multi-channel inputs, yielded substantially inferior results (AUCs: 0.474–0.538), highlighting the limitations of conflating distinct MRI features without modality-specific processing. The primary contribution of this study was a systematic comparison of architectural strategies for differentiating BS and TS using multi-sequence MRI. Our findings reveal a stark divergence in the viability of these strategies. We objectively demonstrated that naively stacking disparate MRI sequences as input channels for a single-branch model was an invalid approach, resulting in a catastrophic failure to learn. In contrast, our results validate two distinct and effective architectural paths: training on a single, informative sequence and integrating all sequences via a multi-branch parallel-processing design. Both of these valid approaches yielded robust and comparable diagnostic performance on the test set (best AUCs of 0.765 and 0.764, respectively), establishing them as successful methods for this clinical problem.

Previous study using machine learning algorithms was developed to differentiate between tuberculous spondylitis and brucellar spondylitis. This model incorporated variables, such as pain severity and MRI features, achieving an AUC of 0.86, indicating high prediction accuracy and clinical efficiency in differential diagnosis [18]. Another study explored CT-based radiomics features combined with machine learning to differentiate between brucellar and pyogenic spondylitis, achieving an AUC of 0.88, demonstrating the potential of radiomics in early and reliable differentiation [19]. Radiomics-based models have shown promise in differentiating between tuberculous TS and BS. A study demonstrated that machine learning models using radiomics and MRI labels could effectively distinguish between these conditions. The study found that support vector machine (SVM) and random forest (RF) models using joint radiomics and MRI labels achieved high areas under the curve (AUCs) of 0.904 and 0.950, respectively, indicating superior performance compared to models using only MRI labels [13]. While effective, these methods rely on manually engineered features, which may not capture the full complexity of the disease pathology. Our deep learning approach offers a distinct potential advantage through its end-to-end learning capability, allowing the models to autonomously discover intricate, hierarchical patterns from the raw imaging data without predefined feature extraction. This reduces operator-dependent variability and holds the potential to identify novel biomarkers. This establishes a clear architectural principle for this clinical problem: parallel processing is superior to simple channel-stacking. The central finding of this study was that architectural design determined whether multi-sequence MRI data could be successfully integrated for the differentiation of brucellar and tuberculous spondylitis. Two architectural strategies—models trained on a single MRI sequence and multi-branch models that processed each sequence in a dedicated parallel stream—both achieved comparable test performance, with AUCs ranging from 0.725 to 0.765. In contrast, the single-branch model that fused T1, T2, and fat-suppressed sequences as RGB-like input channels failed completely, yielding test AUCs between 0.474 and 0.538, consistent with random classification. This failure indicated that naive channelwise stacking was not a suitable approach for this clinical task. The results showed that valid integration of multi-sequence MRI data was achieved by either using a single sequence or a multi-branch architecture; both were functional, whereas single-branch fusion was not. This establishes a clear architectural principle for this clinical problem: parallel processing is superior to simple channel-stacking. However, these methods were limited somehow when compared to CNN architecture when it comes to explanation and end-to-end pipeline development.

The CNN employed in this study was inspired by established deep learning models, such as DenseNet and ResNet, which have proven effective in medical imaging tasks. These models are particularly adept at handling complex image data and extracting meaningful features [20, 21]. The CNN model comprised several convolutional layers, followed by pooling layers, which progressively reduced the spatial dimensions of the input while increasing the depth of the feature maps. This hierarchical approach to feature extraction enabled the model to capture both low- and high-level features essential for distinguishing between BS and TS. Deep convolutional neural networks (DCNNs) have been evaluated for their ability to differentiate between tuberculous and pyogenic spondylitis (PS) using MR images. The performance of the DCNN was comparable to that of skilled radiologists, with an AUC of 0.802, suggesting that deep learning models can be as effective as human experts in this diagnostic task [12]. The input layer received MRI sequences as a three-channel RGB image, where each channel represented one of the MRI sequences (T1, T2, FS). This configuration allowed the model to analyze complementary information from each sequence simultaneously [22]. The complete failure of the single-branch, channel-stacking model warrants specific attention, as it provides a crucial insight into processing multi-modal medical images. This failure likely stems from what can be termed "modality entanglement." Unlike natural RGB images, where channels represent correlated color information, MRI sequences such as T1, T2, and FS represent fundamentally different physical properties of tissues. Forcing a single convolutional stream to learn filters that are meaningful across these disparate contrast mechanisms leads to conflicting gradient updates, hindering the learning process. The model struggles to reconcile T1's anatomical detail with T2's fluid sensitivity and FS's pathology highlighting, resulting in a failure to extract a coherent set of discriminative features. The success of the multi-branch design confirms that allowing each sequence to be processed by a dedicated feature extractor before fusion is essential to preserve and integrate their unique, complementary diagnostic information. However, a key finding from our comparative analysis is that the multi-branch model's increased architectural complexity and use of all three MRI sequences did not translate into a performance advantage over the best-performing single-sequence model. Both approaches achieved comparable test AUCs (approximately 0.76), and the multi-branch model used substantially more input data and trainable parameters. This result factually demonstrates a critical trade-off: while a multi-branch architecture is a valid method for utilizing all available imaging data, it did not offer a corresponding gain in diagnostic accuracy in this study. This suggests that for this specific task, a well-chosen single sequence contained sufficient information for the model to achieve its peak performance.

Single-branch–multi-channel CNNs leveraging multiple MRI sequences consistently outperformed single-sequence models in differentiating medical conditions, primarily due to the richer information provided by multi-sequence data, which enhanced the models' ability to classify and segment medical images with greater accuracy. In this research, we have deployed single-branch model for differentiating BS and TS utilizes a CNN architecture designed to process MRI sequences as an RGB (red–green–blue) three-channel input. This approach leverages the distinct imaging characteristics captured in T1, T2, and FS MRI sequences, which are combined into a single input format like RGB image and were supposed to enhance the model's ability to differentiate between BS and TS, while the result of this single multi-channel approach was disappointing. However, previously published study used CNN–LSTM model utilizing multiparametric MRI sequences demonstrated improved diagnostic outcomes in glioma differentiation, particularly in distinguishing pseudo-progression from true tumor progression, achieving higher accuracy and AUC scores compared to single-sequence CNN models [23]. Similarly, in the diagnosis of focal liver lesions, a 2D DenseNet model employing multi-sequence MRI data slightly outperformed a 3D model, highlighting the influence of model dimensionality and architecture on performance within multi-sequence frameworks [24]. In prostate cancer diagnosis, a 3D CNN trained on multiple MRI sequences achieved AUC scores comparable to those of experienced radiologists, further emphasizing the diagnostic precision enabled by multi-sequence data [25]. For ovarian tumor classification, a multi-instance CNN processing several MRI sequences surpassed traditional radiomics-based methods, showcasing the advantage of deep learning models in handling complex multi-sequence data without manual feature extraction [26] Finally, a CNN trained with seven MRI input channels exhibited superior segmentation performance for head and neck tumors, with specific sequences contributing more significantly to the model’s overall accuracy [27] These findings collectively underscored the critical role of multi-sequence MRI data in enhancing the diagnostic and segmentation capabilities of CNNs across diverse medical conditions. The suboptimal performance of the model likely stemmed from inherent challenges in processing multi-parametric MRI data using conventional single-stream architectures. While RGB-style channel stacking is effective for natural images, where color channels share congruent spatial information, this approach proved problematic for MRI sequences, such as T1, T2, and FS, which provide fundamentally different physical representations of tissue properties. The model's limitations appeared to arise from three key factors: modality entanglement, contrast misalignment, and feature competition. Modality entanglement occurred when early convolutional layers attempted to learn shared filters across sequences with distinct contrast mechanisms. T1 emphasized structural anatomy through longitudinal relaxation times, T2 highlighted fluid content via transverse relaxation, and FS suppressed fat signals to enhance pathological visualization. Forcing a single feature extraction pathway to reconcile these disparate contrast mechanisms likely generated conflicting gradient signals during backpropagation, hindering optimal feature learning for any individual sequence. Contrast misalignment referred to inherent intensity scale discrepancies between sequences. Absolute pixel values in T1 (typically 200–1000), T2 (400–4000), and FS (0–2500) existed on different numerical ranges despite clinical normalization. Simple linear normalization to [0–255] preserved relative intra-sequence relationships but discarded critical inter-sequence diagnostic patterns, such as the specific T2 hyperintensity thresholds differentiating TS's caseous necrosis from BS's inflammatory edema. Feature competition emerged when diagnostically critical features from one sequence were diluted by less discriminative patterns from others. This indicated an architectural bias toward higher contrast sequences rather than learned integration of complementary information. These challenges underscored the need for specialized architectural components when fusing multi-sequence MRI data, such as modality-specific normalization layers, cross-sequence attention mechanisms, or differential learning rates per channel—strategies conspicuously absent in standard CNN architectures applied to concatenated inputs. In clinical interpretation of CAM outputs, the most frequent regions of diagnostic focus for brucellar spondylitis BS and TS on MRI are distinct and clinically meaningful. BS predominantly involves the lumbar spine, with CAM highlighting vertebral bodies that show mild destruction, pronounced vertebral hyperplasia, and limited abscess formation. These lesions typically spare the intervertebral discs and rarely cause significant kyphosis or dead bone, reflecting a more localized and less aggressive inflammatory process. In contrast, TS is more frequently centered in the thoracic spine, with CAM outputs emphasizing areas of severe vertebral destruction, vertebral collapse, marked kyphotic deformity, and extensive abscesses—especially paraspinal and psoas abscesses. TS often affects multiple contiguous vertebrae, with greater involvement of the posterior vertebral elements and a higher risk of spinal cord compression. These imaging patterns, as revealed by CAM, align with established radiological distinctions: BS is characterized by localized lumbar involvement and hyperplastic changes, while TS demonstrates widespread thoracic destruction and aggressive soft tissue extension. This differentiation provides a robust, explainable basis for deep learning models to assist clinicians in distinguishing between BS and TS in practice [2, 28].

Multi-branch CNNs, when leveraging multiple MRI sequences, consistently outperformed single-branch models in differentiating various medical conditions. In our research, we also discovered multi-branch model outperformed compared to single-branch multi-channel model. This success was primarily attributed to their ability to process and integrate multiple MRI data types simultaneously, which enhanced diagnostic accuracy. For example, a study on multiple sclerosis lesion segmentation demonstrated that multi-branch CNNs, by encoding information from multiple modalities, significantly improved segmentation performance compared to models using only a single sequence [29]. In a similar context, multi-branch CNNs also achieved superior accuracy and specificity in glioblastoma differentiation when compared to single-branch models [30]. On the other hand, single-branch CNNs, which typically process only one MRI sequence at a time, were often limited in their capacity to capture the full spectrum of diagnostic information. This was evident in a brain tumor segmentation study, where single-sequence CNN models underperformed relative to multi-sequence models trained with a broader data set [31]. Furthermore, the integration of various MRI sequences allowed multi-branch CNNs to provide more comprehensive and accurate diagnostic results, as demonstrated in tasks, such as prostate cancer classification and nasopharyngeal carcinoma segmentation [32]. Overall, the literature shows that multi-branch CNNs are a frequently employed architecture for synthesizing information from multiple MRI sequences, with the goal of improving diagnostic precision across a wide range of conditions.

## Conclusion

Our systematic comparative analysis demonstrates that the architectural strategy for integrating multi-sequence MRI data is a critical factor in differentiating brucellar from tuberculous spondylitis. We have established that naive channelwise fusion is an invalid approach, leading to model failure. In contrast, this study validates that both single-sequence models and multi-branch parallel-processing models are effective strategies, achieving comparable and robust diagnostic performance. This work clarifies the fundamental architectural principles for this diagnostic task, showing that while successful integration of all sequences is possible via a multi-branch design, it does not necessarily offer a performance advantage over a well-optimized model trained on a single, informative sequence. Our explainability analyses through various Grad-CAM techniques provide valuable insights into model decision-making processes, enhancing transparency and potentially facilitating clinical adoption. These visualizations allow radiologists to understand which image regions influence model predictions, representing an important step toward responsible AI implementation in clinical settings. While further validation through prospective and multi-center studies is necessary, our findings establish a foundation for developing AI-assisted diagnostic tools for spinal infections. Implementation of such tools could reduce diagnostic delays, improve treatment planning, and ultimately enhance patient outcomes in the management of these challenging conditions.

## Limitations

This study has several limitations, which we frame in the context of our specific scientific objective. The primary goal of this work was to conduct a foundational comparative analysis to identify valid deep learning architectures for this specific clinical problem, rather than to develop a universally generalizable clinical tool. First and foremost, this study was conducted using a retrospective data set from a single center. While this inherently limits the direct generalizability of our performance metrics (e.g., AUC), this controlled data environment was intentionally used to achieve our primary objective. It allowed us to effectively decouple the question of architectural performance from the confounding variable of data source heterogeneity (e.g., variations in scanner manufacturers, field strengths, and imaging protocols). By doing so, we could rigorously validate that naive sequence-stacking is an invalid architectural choice, while parallel-processing multi-branch models are a valid one. Establishing this architectural blueprint is a critical prerequisite that can now inform the design of future, more robust multi-center validation studies. Second, the sample size of 235 patients, while substantial for this specific rare spinal infection, remains relatively modest for deep learning. This limitation, dictated by the rarity of Brucellar and Tuberculous Spondylitis, may restrict the models' ability to learn the full spectrum of disease presentation and could be a contributing factor to the modest performance observed. Future work leveraging federated learning or large-scale multi-institutional collaborations will be necessary to overcome this barrier. Third, this study was designed as a binary classification problem between BS and TS. This does not reflect the full clinical challenge, which involves a broader differential diagnosis, including pyogenic spondylitis, spinal tumors, or fungal infections. Future work should expand these validated architectures to a multi-class classification framework to increase clinical utility. Finally, while we incorporated explainability techniques (CAMs), their direct clinical relevance requires formal validation by radiologists to ensure the models' "attention" aligns with established diagnostic criteria. The known variability of CAM heatmaps also warrants the exploration of more robust XAI methods in subsequent studies. Despite these limitations, this study successfully establishes an architectural blueprint for future research aimed at developing and validating generalizable AI tools for spinal infection differentiation.

## Supplementary Information

Additional file1 (PDF 1051 kb)

## Data Availability

Upon a reasonable request, the corresponding authors of this article will provide unrestricted access to the original data.
