# Supplementary material for: Comprehensive comparative analysis of explainable deep learning model for differentiation of brucellar spondylitis and tuberculous spondylitis through MRI sequences
Source: Eur J Med Res. 2025 Dec 24;31:146. doi: 10.1186/s40001-025-03731-9 (PMC12849075; doi:10.1186/s40001-025-03731-9)
Supplement: Supplementary file 1 — Additional file1 (PDF 1051 kb) [file 40001_2025_3731_MOESM1_ESM.pdf]

## Supplementary Table 1 Summary of MR Protocols

| Manufacturer | Scanner | FS | Sequence | TR | TE | FOV | Matrix | Thickness | Spacing |
|---|---|---|---|---|---|---|---|---|---|
| Siemens | MAGNETOM Aera | 1.5T | T1WI | 520 ms | 8.4 ms | 300 mm | 256 × 256 | 4 mm | 1 mm |
| | | 1.5T | T2WI | 3000 ms | 94 ms | 300 mm | 256 × 256 | 4 mm | 1 mm |
| | | 1.5T | FS-T2WI | 3700 ms | 85 ms | 300 mm | 256 × 256 | 4 mm | 1 mm |

FS: Field Strength; TR: Repetition Time; TE: Echo Time; FOV: Field of View.

## Supplementary Table 2 Summary of Deep Learning Image Models with Input Image Size and Pretrained Weights

| Models | Input Image Size | Initial Weights Dataset |
|---|---|---|
| ResNet152 | 224x224 | IMAGENET1K_V2 |
| DenseNet121 | 224x224 | IMAGENET1K_V1 |
| MobileNetV3 | 224x224 | IMAGENET1K_V2 |
| ShuffleNetV2 | 224x224 | IMAGENET1K_V1 |
| VGG19 | 224x224 | IMAGENET1K_V1 |

# Appendix S1

**Baseline model** optimization process utilized the Adam optimizer with a learning rate of 1e-4, coupled with a *ReduceLROnPlateau* scheduler to dynamically reduce the learning rate by a factor of 0.5 when the validation loss plateaued. The scheduler had a patience of one epoch, a cooldown of one epoch, and a minimum learning rate threshold of 1e-8. Cross-entropy loss was employed as the objective function to minimize the categorical classification error. Training was conducted for a maximum of 200 epochs per model, with a validation interval of one epoch to evaluate performance metrics and save the best-performing model. Model evaluation during training focused on the area under the receiver operating characteristic curve (AUC) and accuracy, with the AUC serving as the primary metric for selecting the best model. For each validation step, predictions were aggregated, and metrics were computed using MONAI's ROCAUCMetric. To ensure reproducibility and robustness, models were trained separately on T1, T2, and FS MRI datasets. Validation loss was monitored at each epoch, and the best model weights were saved whenever an improvement in AUC was observed. Additionally, GPU memory was cleared after each training run using PyTorch's torch.cuda.empty_cache() method, and garbage collection was invoked to optimize resource utilization. All training results, including loss, AUC, and accuracy values across epochs, were stored for subsequent analysis. This systematic approach ensured rigorous training and evaluation of all models across all MRI modalities, with detailed parameters and results provided in the supplementary materials for transparency and reproducibility.

# Appendix S2

The dataset for **single-branch** model was prepared by collecting T1, T2, and FS MRI sequences from clinical cases, with each sequence preprocessed to ensure consistency. Images were resized to 224×224 pixels, and data augmentation was performed using MONAI's transformation utilities. Augmentation steps included random spatial cropping to maintain dimensions, random rotation within ±15 degrees, horizontal flipping with a probability of 50%, and random zooming between 90% and 110% of the original size. The dataset was divided into training (70%), validation (15%), and testing (15%) sets using stratified sampling to maintain class balance. A batch size of 64 was used for all data loaders. Pretrained models initialized with ImageNet weights were modified to accept three-channel inputs by replacing the first convolutional layer with one that processes three channels. The final fully connected layers were adjusted to output predictions for two classes. Cross-entropy loss was used as the objective function, and the Adam optimizer was employed with an initial learning rate of 1e-4. A ReduceLROnPlateau scheduler was applied to dynamically reduce the learning rate by a factor of 0.5 when validation loss plateaued for one epoch. Training was conducted for 200 epochs per model, and validation was performed at the end of each epoch. The best-performing model weights were saved based on the highest area under the receiver operating characteristic curve (AUC) score during validation. Training metrics, including loss, AUC, and accuracy, are detailed in the supplementary material for reproducibility.

# Appendix S3

To train and evaluate the **Multi-Branch** Model, we prepared a dataset comprising T1, T2, and FS MRI sequences, ensuring that all three modalities were available for each case. The dataset was split into training, validation, and test sets with a 0.7:0.15:0.15 ratio, maintaining class balance through stratified sampling. Preprocessing included resizing images to 224×224 pixels, applying data augmentation (random rotation, flipping, cropping, and zooming), and normalizing pixel intensities. The training process used a batch size of 32 and employed the Adam optimizer with a learning rate of 1e-4. A ReduceLROnPlateau scheduler was applied to dynamically adjust the learning rate based on validation loss. Cross-entropy loss was used as the objective function to minimize classification error. The model was trained for 200 epochs on an NVIDIA GPU, with early stopping implemented to prevent overfitting. Performance metrics, including AUC and accuracy, were calculated on the validation set at each epoch. The best-performing model, determined by the highest validation AUC, was saved for further evaluation on the test and external datasets. All training and evaluation scripts were implemented in PyTorch, and the code is available upon request.

# Appendix S4

The training and evaluation of the Comparative Multi-Branch Model were carried out on a multi-sequence MRI dataset, ensuring that each case included all three modalities (T1, T2, FS). The dataset was divided into training, validation, and test sets with an 70:15:15 split, maintaining class balance using stratified sampling. Data preprocessing included resizing images to 224×224 pixels, applying data augmentation techniques such as random rotation (±15°), flipping, cropping, and zooming (scaling range: 0.9–1.1). The Adam optimizer was used with a learning rate of 1e-4, and a *ReduceLROnPlateau* scheduler was employed to adjust the learning rate dynamically based on validation loss. Cross-entropy loss was used as the objective function. The models were trained for 300 epochs with a batch size of 32 on an NVIDIA GPU, and early stopping was implemented to prevent overfitting. Performance metrics such as AUC and accuracy were computed on the validation set at each epoch to monitor progress. The best-performing model for each backbone architecture was determined based on the highest validation AUC and subsequently evaluated on internal and external test datasets. All details of the training pipeline, including the source code, are provided in the supplementary materials to ensure reproducibility.

**Supplementary Table 3 Demographic data of enrolled population**

| Characteristics | All (n = 235) | TS (n = 153) | BS (n = 82) |
|---|---|---|---|
| **Age (years), mean ± SD** | 47.1 ± 20.0 | 47.1 ± 20.1 | 47.1 ± 19.9 |
| **Gender, n (%)** | | | |
| Female | 144 (61.3%) | 99 (64.7%) | 45 (54.9%) |
| Male | 91 (38.7%) | 54 (35.3%) | 37 (45.1%) |

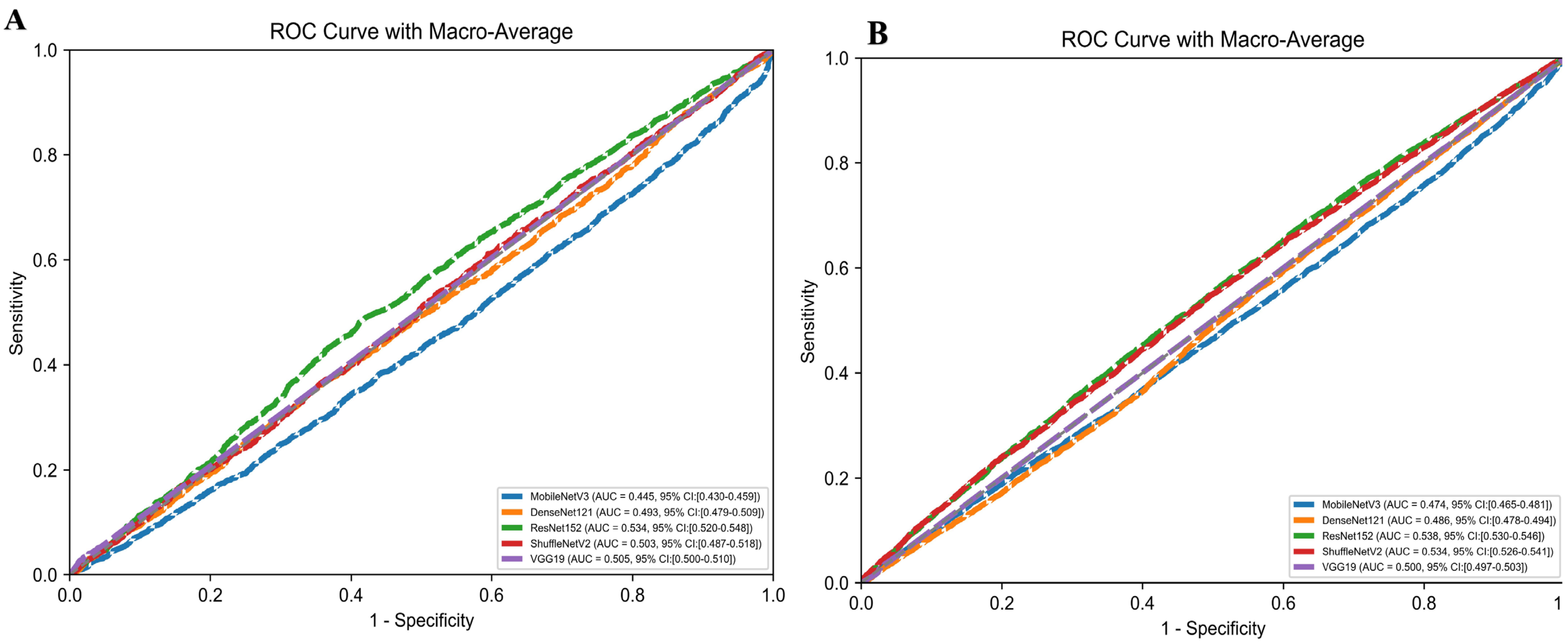


**Supplementary Fig. 1**: ROC Curve validation of single-branch model

(A): Receiver operating characteristic (ROC) curves illustrating the comparative performance of the five selected model architectures (MobileNetV3, DenseNet121, ResNet152, ShuffleNetV2, and VGG19) on the internal validation dataset for differentiating brucellar from tuberculous spondylitis; (B): Corresponding ROC curves for the same five models evaluated on the internal testing dataset, providing an assessment of their generalization capabilities on unseen data from the same source population. Each curve represents a different model, with the legend providing the calculated Area Under the Curve (AUC) and its 95% confidence interval.

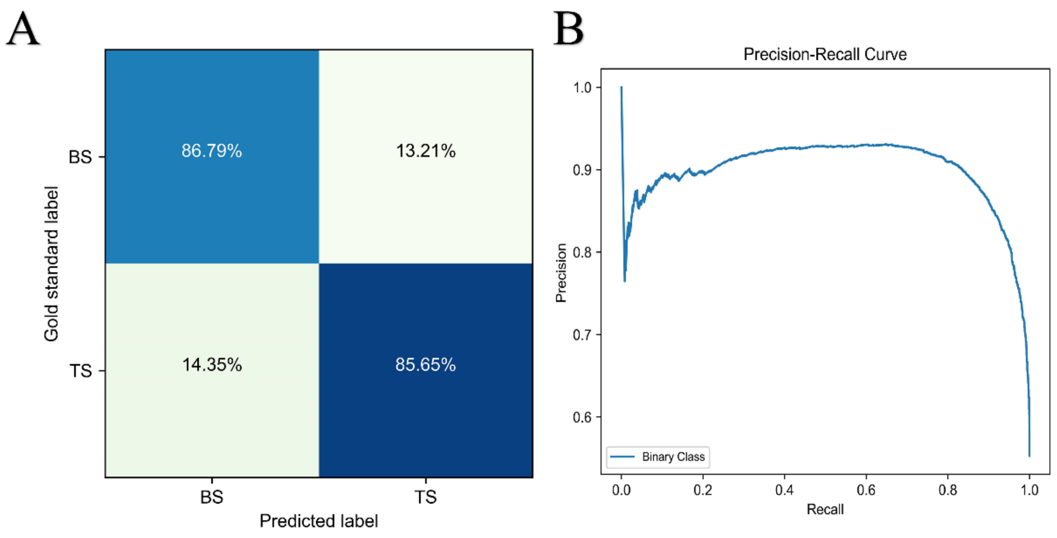


**Supplementary Fig. 2**: Confusion matrix and precision-recall curve of ShuffleNetV2 (A): A confusion matrix is presented, demonstrating the model's classification accuracy for both BS and TS. The matrix provides a detailed breakdown of true positives, true negatives, false positives, and false negatives, offering a clear view of how well the model distinguishes between the two sample types. (B): A precision-recall curve is shown, which illustrates the trade-off between precision (the accuracy of positive predictions) and recall (the ability to identify all relevant instances) across various classification thresholds. This curve helps in understanding the model's performance in scenarios where the balance between precision and recall is critical, such as in imbalanced datasets.
